# Protective Effect of *Medicago sativa* L. (Fabaceae) against Excision and Incision Wound Models in Diabetic Rats

**DOI:** 10.2174/0115733998345972250117103918

**Published:** 2025-02-28

**Authors:** Phool Chandra, Mohammad Kaleem, Neetu Sachan, Anurag Verma, Atul Kabra

**Affiliations:** 1 Department of Pharmacology, Teerthanker Mahaveer College of Pharmacy, Teerthanker Mahaveer University, Moradabad, U.P, 244001, India;; 2 School of Pharmaceutical Sciences, IFTM University, Lodhipur Rajput, Delhi Road (NH-24), Moradabad, U.P, 244102, India;; 3 Maharana Pratap College of Pharmacy, Mandhana, Kanpur, U.P, 209217, India;; 4 Department of Pharmaceutics, Teerthanker Mahaveer College of Pharmacy, Teerthanker Mahaveer University, Moradabad, U.P, 244001, India;; 5 University Institute of Pharma Sciences, Chandigarh University, Mohali, Punjab, 140301, India

**Keywords:** *Medicago sativa*, alfalfa, diabetic rats, wound healing, excision wound, incision wound

## Abstract

**Introduction:**

*Medicago sativa* (*M. sativa*), commonly known as Alfalfa, is a herb from the Fabaceae family that has a long history of being used to treat digestive, diabetic, and blood disorders, as well as to support liver health. The objective was to evaluate the effects of ethanolic extract of *M. sativa* (EEMS) on wounds in normal rats or alloxan hydrate-induced diabetic rats.

**Methods:**

The wounds were created by excision (n=30) and incision (n=30) in rats. The Group II-V were diabetic rats treated with simple ointment BP, 10% weight-based povidone-iodine (10% PI), ointment of 5% w/w EEMS (5% w/w OEEMS), and 10% w/w EEMS (10% w/w OEEMS). Group 1 acted as a control and was treated with simple ointment BP. The wound area in the diabetic control groups was 292.33 ± 0.8 mm^2^ on the 18^th^ day.

**Results:**

Rats treated with 10% PI, 5% OEEMS, and 10% OEEMS showed a significant reduction in wound area of 68.33 ± 1.29, 248.33 ± 1.30, and 61 ± 1.91 mm^2^, respectively, on the 18^th^ day as compared to the control group. Rats treated with 10% PI, 5% w/w OEEMS, and 10% w/w OEEMS showed a significant increment in wound-breaking strength, respectively, as compared to diabetic rats on day 10 in the incision wound model.

**Conclusion:**

The results demonstrated that the OEEMS has potent wound-healing properties in diabetic rats.

## 1 INTRODUCTION

Diabetes mellitus, a chronic metabolic disorder characterized by hyperglycemia, has far-reaching effects on various physiological processes, including wound healing [1, 2]. Wound healing is a complex and dynamic process involving a series of coordinated events that aim to restore tissue integrity and function [3]. However, individuals with diabetes often face compromised wound-healing abilities, leading to delayed recovery and increased susceptibility to chronic wounds [4]. The impaired wound-healing process in diabetes is attributed to factors, such as reduced blood flow, neuropathy, immune dysfunction, and altered cellular responses [2, 5].

The Fabaceae family includes therapeutic plants like alfalfa (*Medicago sativa*). It has a long history of being used as a Homoeopathic and Ayurvedic medical method to treat many disorders, including those affecting the central nervous system and digestive systems [6]. *Medicago sativa* is utilized as a culinary ingredient in China, North Africa, Russia, and the United States because of its abundant vitamins and bioactive components [7]. The product contains a range of enzymes that assist in digestion, including amylase, invertase, and pectinase [8]. Research in pharmacology has demonstrated that *Medicago sativa* extract possesses various beneficial properties. These include antidiabetic [9, 10], antioxidant [11, 12], anti-inflammatory [13], antifungal [14], cardio-protective [12], antimicrobial [15, 16], anticancer [17], hepatoprotective [18], anti-coagulant, hypocholesterolemic [19], and anti-scorbutic effects [20], and potential therapeutic applications in central nervous system (CNS) disorders [21]. Phytochemical study in literature has revealed that *Medicago sativa* contains flavonoids [22], alkaloids [23], phytoestrogens [24], phytosterols [25], amino acids [26], coumarins [27], organic acids [26], phenolic compounds [28], proteins [29], vitamins [30], digestive enzymes [31], triterpenes, and saponins [32-34]. Alfalfa tea is utilized for enhancing the digestive system. Diabetics utilize sprouts derived from seeds [35].

As researchers seek innovative approaches to enhance wound healing in diabetic patients, the evaluation of pharmacological interventions becomes imperative. The exploration of *Medicago sativa* with potential wound-healing properties presents a promising avenue to mitigate the challenges associated with impaired healing in diabetes. This study focuses on assessing the wound-healing activity of EEMS in diabetic rats.

## 2 MATERIALS AND METHODS

### 2.1 Cultivation and Collection

The seeds were procured from the local market of Khari Baoli, Delhi. The plant was cultivated in a field located in Amroha, Uttar Pradesh, India, on rich, friable, well-drained, loamy soil. The land was thoroughly ploughed before sowing. Seeds were soaked in water overnight to enhance germination and then sown in the month of November. Germination occurred within 2 to 3 weeks, followed by thinning of seedlings. Flowering commenced in the first week of February, after which the plants were harvested and shade-dried.

### 2.2 Identification and Authentication

The plant sample was gathered and verified by Dr. Ashok Kumar, Head of Department of Botany, IFTM University, Moradabad. The sample was stored in the Department of Botany with the reference number 2019/SOS/BOT/73.

### 2.3 Preparation of Ethanolic Extract of *Medicago sativa* (EEMS)

The aerial parts of *Medicago sativa * were collected, dried in the shade, and pulverized. The powdered drug 300 g was soaked in petroleum ether for 24 hours at room temperature. After filtration with muslin fabric, the residue was treated with ethanol for 24 hours. The obtained ethanol extract was dried and concentrated under a rota vacuum evaporator. The percentage yield for the extract was determined by using the following formula:


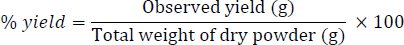


### 2.4 Preliminary Phytochemical Screening

Ethanol extract obtained from *M. sativa* was subjected to preliminary phytochemical screening by employing standard chemical tests for the determination of phyto-constituents [36].

### 2.5 Drugs and Chemicals

The chemicals utilized were: Diethyl ether (Central Drug House (P) Ltd New Delhi), Betadine ointment (Povidone-iodine 10% w/w) (Win-Medicare Pvt. Ltd, New Delhi), alloxan monohydrate (Sisco Research Laboratories Pvt. Ltd. New Delhi), formalin, wool fat, hard paraffin, cetostearyl alcohol, and white soft paraffin.

### 2.6 Animals

Wistar albino rats, weighing between 120-200 g of either sex, were procured from the university's animal facility. The rats were housed in a room with a temperature of about 25 ± 2°C, a relative humidity of 50-55%, and a 12:12 hrs light and dark cycle. Animals were given unrestricted access to normal food for rodents. The investigations were conducted in compliance with the guidelines of the Institutional Animal Ethics Committee (IAEC) governed by CPCSEA, India, under resolution No. 2019/837ac/M.Pharm./04. An orogastric cannula of standard size was utilized for the purpose of administering drugs orally [37, 38].

### 2.7 Induction of Diabetes to the Animals

Diabetes was induced by intraperitoneal injection of alloxan hydrate at a dosage of 140 mg/kg. The animals were placed under surveillance. The blood glucose level was assessed prior to and 72 hours following alloxan administration to verify the onset of diabetes. The diabetic animals were rendered stable for a period of five days, after which the experiment commenced on a subsequent day, referred to as day 0. Only animals with blood glucose levels exceeding 13.89 mmol/L were selected and utilized for the investigation [39].

### 2.8 Preparation of Ointment

The preparation of simple ointment was conducted as per British Pharmacopoeia, consisting of hard paraffin, white soft paraffin, wool fat, and cetostearyl alcohol. The composition of simple ointment is given in Table **1** [40].

The simple ointment base was created by melting the hard paraffin to which other ingredients were added in decreasing order of their melting temperature. The melting was achieved on a water bath under constant stirring till a homogenous consistency was achieved. The concoction was taken off the heat and agitated until it reached a cool temperature. To create ethanol extract ointment, 1.25 g and 2.5 g of the extract were mixed with a small quantity of simple ointment base to produce 5% w/w and 10% w/w ointments, respectively, by levigation method. The remaining portion of the uncomplicated ointment base was then added gradually and carefully blended. Ultimately, the ointment extract was moved to a sterile container for the purpose of applying it to the skin during the experiment.

### 2.9 Excision Wound Model

The study was conducted in rats divided into five groups (n=6) as described below for developing excision wounds:

**Group I:** Animals treated with simple ointment BP and served as control.

**Group II:** Animals treated with simple ointment BP and served as diabetic control.

**Group III:** Animals treated with povidone-iodine (10% w/w PI) and served as the reference drug for comparison.

**Group IV:** Animals treated with 5% w/w OEEMS ointment.

**Group V:** Animals treated with 10% w/w OEEMS ointment.

The animals were administered anesthesia before and during the process of creating wounds. The rats were subjected to excision wounds following the methodology outlined by Morton and Melone, with certain modifications. The animal's dorsal fur was removed using an electric clipper, and the intended wound region was marked on the animal's back with methylene blue using a circular steel stencil. A circular region of 500 mm^2^ of a length of 2.5 cm and a depth of 0.2 cm was excised to create an open wound using scissors and a surgical blade. The groups were then treated until full epithelialization occurred [41].

#### 2.9.1 Measurement of Wound Area and Wound Contraction

The wound area measurement was done at predetermined time points (days 2, 4, 6, 8, 10, 12, 14, 16, and 18) by delineating the wounds and measuring using a geometric divider and geometric scale. The assessment involved measuring the changes in the size of the wound, which provided information about how quickly the wound was shrinking and the time it took for new skin to form. The proportion of wound contraction was determined using the following formula:


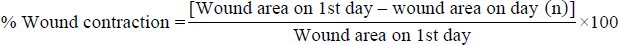


Where n represents the number of days, such as the 2^nd^ or 4^th^ day.

#### 2.9.2 Determination of the Period of Epithelialization

The detachment of the scab from the wound is regarded as an indication that the wound has healed or as the final stage of full epithelialization. The duration necessary for this process is referred to as the epithelialization period.

### 2.10 Incision Wound Model

For developing incision wounds and conducting a wound closure study, again, 30 rats were divided into five groups (n=6) treated as described above. The rats in the model above were given anesthesia before and during the wound formation. An electric clipper was used to shave the animals' dorsal fur. A 6 cm long incision was made down the side of the spine, cutting through the skin and underlying muscle. Following the incision, surgical sutures were used to close the separated skin at regular intervals of 1 cm, and the wound remained exposed. On the 8^th^ day of injury, removal of suture and continuation of treatment was done. The assessment of tensile strength was followed on the 10^th^ day after the injury [41].

#### 2.10.1 Determination of Wound-breaking Strength

The study was conducted using an assembly comprising immovable shelves on a table and two Allis forceps. One was secured opposite to the side of the shelf while the other was fastened to a rope and connected to an empty bag to which weight was added. After 10 days of being wounded, each rat was sedated with diethyl ether in order to immobilize the animal. The forceps were then securely positioned 1 cm distant from the repaired tissue of the incised skin area and aligned in a parallel manner. Tap water was permitted to enter the bag through an intravenous (i.v.) line. An incremental gain in weight was transferred to the bag, causing the wound edges to separate. Upon the immediate appearance of a gaping wound, the flow of water was promptly halted, and the increased weight was recorded as a quantitative measure of the breaking strength in grams [40].

### 2.11 Statistical Analysis

The data were presented as the mean value plus or minus the standard error of the mean (n=6). The statistical difference was assessed by one-way analysis of variance (ANOVA) using Dunnett's test. The results exhibited a statistically significant result with a *p*-value below 0.05. The statistics were conducted utilizing the Graph-Pad Prism software.

## 3 RESULTS

### 3.1 Yield

The yield was as follows: total weight of dry powder = 300 (g); Observed yield = 107.6 (g). Therefore, the yield was found as 35.86%.

### 3.2 Preliminary Phytochemical Analysis

The findings of the initial phytochemical analysis were recorded as alkaloids, carbohydrates, flavonoids, phenolic compounds, phytosterols, and saponins (Table **2**).

### 3.3 Excision Wound Model

Mean blood glucose levels in the normal group were 4.88 ± 0.16 mmol/L on day 0 to 4.74 ± 0.08 mmol/L on day 18^th^, without much variation during the study shown in Table **3**. In the diabetic control group, there was a gradual increase in blood glucose levels from the mean value of 21.95 ± 0.36 mmol/L on day 0 to 24.53 ± 0.33 mmol/L on day 10^th,^ and it was found to decrease in blood glucose level to 23.62 ± 0.42 mmol/L on 18^th^ day. In test groups, less increment was found in blood glucose levels from day 0 to day 10^th^ of the experiment. There was a gradually increasing reduction in blood glucose level on the 18^th^ day of the experiment, which was statistically significant compared to diabetic control.

#### 3.3.1 Effect on Body Weight, Food, and Water Consumption Levels

The administration of alloxan led to a statistically significant (*p* <0.05) increase in food and water intake, as well as a decrease in body weight levels, in the diabetes control group compared to the normal control group (Table **4**). Moreover, a significant ameliorative effect was observed in the OEEMS 5% w/w treated group. In addition, the administration of Povidone iodine and OEEMS 10% w/w treated groups also demonstrated a notable (*p* < 0.05) improvement in body weight, fluid intake, and food consumption levels compared to control animals.

#### 3.3.2 Contraction in Wound Area (mm^2^) and % Wound Contraction

The application of ointments containing ethanolic extracts of *M. sativa* had a notable impact on the wound-healing process in diabetic rats. The wound contraction progress resulting from the application of 5% w/w and 10% w/w ointments containing ethanolic extracts, a simple ointment base, and Povidone iodine 10% (w/w) ointment is displayed in Figs. (**1**-**4**). On days 0, 4, 8, 12, 16, and 18, the wound area (mm^2^) was measured in all studies of animals. Wound area in the diabetic control group was 542.66 ± 1.42, 525.66 ± 0.6, 479 ± 0.34, 422.66 ± 0.84, 345.33 ± 0.98, and 292.33 ± 0.8 mm^2^ on respective days. Rats treated with 5% OEEMS showed a significant reduction in wound area on the 12, 14, 16, and 18 days. (376 ± 1.03, 346.66 ± 0.4, 299.33 ± 0.6 and 248.33 ± 1.3 mm^2^ *p* < 0.001) as compared to diabetic control. Whereas treatment with Povidone-iodine (PI 10%) showed a significant reduction in wound area on days 8, 10, 12, 14, 16, and 18 (398.33 ± 0.6, 342.33 ± 1.2, 292 ± 1.15, 213.66 ± 0.95, 129.66 ± 0.8 and 68.33 ± 1.29 mm^2^ *p*<0.001) than diabetic control group. Treatment with 10% OEEMS also showed significant reduction in the wound area (496.66 ± 0.66, 441.66 ± 0.61, 393.33 ± 0.98, 340.66 ± 0.98, 286 ± 1.4, 209.33 ± 0.84, 124 ± 0.73 and 61 ± 1.91 mm^2^ *p* <0.001) on days 4, 6, 8, 10, 12, 14, 16, and 18, as compared to the control group, as well as all study treatments (Table **5**).

#### 3.3.3 Period of Epithelialization

The epithelialization period observed in OEEMS (10% w/w and 5%w/w) and standard (PI 10% w/w) drug was found to be 19.16 ± 0.6 days, 23.66 ± 0.49 days and 19.83 ± 0.3 days, respectively. The complete epithelialization was observed on the same day of OEEMS (10%w/w) and standard drug; hence, diabetic control rats took 4 days to increase the period of epithelialization, *i.e.*, 26.83 ± 0.65 days when compared to normal control 22.66 ± 0.42 days (Table **6**).

### 3.4 Incision Wound Model

Table **7** indicates that the average blood glucose levels in the normal group ranged from 4.64 ± 0.19 mmol/L on day 0 to 5.07 ± 0.12 mmol/L on day 10, with minimal fluctuations throughout the research. In the diabetic control group, there was a progressive rise in blood glucose levels, starting from the average value of 18.19 ± 0.13 on day 0^th^ to 20.55 ± 0.26 on day 10^th^. In the standard group, both 5% and 10% w/w OEEMS showed a very less increment in blood glucose level from day 0^th^ to day 10^th^.

#### 3.4.1 Wound Breaking Strength

The application of ointments containing ethanol extracts of *M. sativa* had a notable impact on the strength of wound healing in diabetic rats. The wound-breaking strength progress resulting from the application of 5% w/w and 10% w/w ointments containing ethanolic extract, simple ointment base, and povidone-iodine 10% w/w ointment is displayed in Table **8**. The application of ointments containing ethanolic extracts of *M. sativa* had a notable impact on the strength of wound healing in diabetic rats. On day 10^th^, the wound-breaking strength (g) was measured in all studies of animals. Wound-breaking strength in the diabetic control group was 432.5 ± 4.47 g. Normal control rats showed a significant increment in wound-breaking strength (524.66 ± 6.5 g) as compared to the diabetic control. Rats treated with 5% OEEMS showed a significant increment in wound-breaking strength (498.83 ± 5.31 g, *p* < 0.001) as compared to diabetic control. Whereas, treatment with Povidone-iodine 10% w/w showed a significant increment in wound breaking strength on day 10 (585.83 ± 3.33 g, *p* <0.001) compared to the diabetic control group. Treatment with 10% w/w OEEMS also showed a significant reduction in the wound-breaking strength area (596.66 ± 2.29 g, *p* <0.001) on day 10, as compared to the diabetic control group as well as all study treatments.

## 4 DISCUSSION

A wound is characterized by the disruption or damage to the cellular, anatomical, or functional integrity of living tissues. It is a well-established medical condition that has been present throughout human history and is widely recognized as a significant challenge in clinical treatment. The healing process of wounds is facilitated by a complex interplay of blood cells, cytokines, and growth factors. This sophisticated network ultimately results in the restoration of the wounded skin or tissue to its original state [42].

Wound healing is an intricate process that involves a diverse range of cellular and matrix components working together to restore the integrity of the damaged tissue. The intricacy of the healing process can be condensed into four overarching categories that align with the chronological order of normal healing: hemostasis, inflammation, cell proliferation, and tissue formation, which collectively establish an initial hemostatic plug to safeguard the wound [43]. Neutrophils migrate to the wound site within 24 hours and remove cellular waste, foreign substances, and germs. Within a span of 2-3 days, the population of inflammatory cells undergoes a transition, with macrophages taking over and fibroblasts emerging at the site of the wound. Within a period of 3 to 5 days, the fibroblasts undergo activation and initiate the process of collagen synthesis. During the formation of the collagenous matrix, tightly packed fibers fill the wound site. As the wound undergoes remodeling, it gradually gains strength over time [44].

Diabetes mellitus leads to compromised and delayed wound healing due to the intricate nature of the wound-healing process. Diabetes mellitus can lead to elevated blood glucose levels, which may cause damage to the endothelial cells and potentially block small blood arteries. Additionally, high blood sugar levels can affect the activity of white blood cells, reducing their ability to move towards and engulf foreign particles. This can result in impaired wound healing and an increased susceptibility to infections [45]. Multiple studies have shown that plant products have the potential to be beneficial in promoting wound healing. They are often selected due to their lack of undesirable side effects and their high level of effectiveness [46].

Alloxan-induced diabetic rats exhibited heightened food and water intake, as well as reduced body weight. This suggests a state of polyphagia and weight loss caused by an increased degradation of tissue proteins [47]. Prior research has indicated that the reduced body weight observed in diabetic rats may be attributed to both dehydration and the breakdown of lipids and proteins. Research has demonstrated a statistically significant (*p* <0.05) elevation in blood glucose levels in the diabetic group as compared to the normal control group. There was a significant rise in blood glucose levels in all the treatment groups on the 10^th^ day of the trial, followed by a drop on the 18^th^ day. Two models, namely the excision and incision wound models, were employed to evaluate the wound healing properties of ethanolic extract of *M. sativa* in diabetic rats. The findings of the current study demonstrated that *M. sativa* exhibits a distinct pro-healing effect.

The findings of our study demonstrate that elevated blood sugar levels negatively affected the healing of wounds in the dorsal region of rats. OEEMS expedites the process of wound healing. The herb *Medicago sativa* is commonly utilized for its antibacterial and anti-inflammatory qualities. Thus, the study was conducted thoroughly to examine the combined wound healing effects of herb extract indicated before. The topical application of a formulation blend was compared to a commercially available Povidone-iodine ointment in rats with excision and incision wounds.

In the excision wound model, non-diabetic control rats exhibited accelerated healing compared to the diabetic control rats. Both dosages of OEEMS resulted in a considerable increase (*p* <0.001) in wound contraction, a significant (*p* <0.001) enhancement in the rate of % wound contraction, and an extremely significant (*p* <0.001) increase in the period of epithelialization compared to the diabetic control rats. Therefore, it may be concluded that OEEMS has a beneficial effect on healing, as demonstrated by the aforementioned data.

For the incision wound repair study, OEEMS was observed to increase the breaking strength of the wound, suggesting higher collagen concentration and fiber stabilization [48]. Since incision wounds treated with 5% w/w and 10% w/w OEEMS showed greater wound-breaking strength, it may be speculated that it not only increases collagen synthesis per cell but also facilitates protein cross-linking.

## CONCLUSION

The ethanolic extracts of *M. sativa* demonstrate enhanced wound healing activity in alloxan-induced diabetic rats compared to the diabetic control rats. Further, there was an increase in wound contraction and wound-breaking strength. Therefore, this study is superior in terms of efficacy and safety compared to other medications. This is primarily due to the increase in population, insufficient availability of drugs, high cost of treatments, adverse effects of various allopathic drugs, and the need to alleviate the financial burden on developing nations. Consequently, there is a growing emphasis on utilizing plant materials as a medicinal resource for human ailments.

## Figures and Tables

**Fig. (1):**
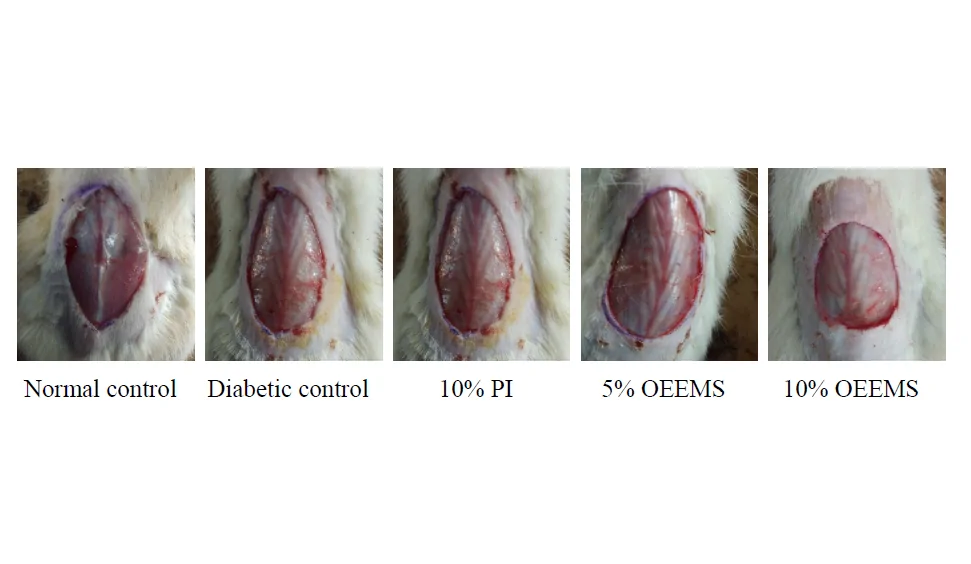
Representative photograph showing a wound on initiation day.

**Fig. (2):**
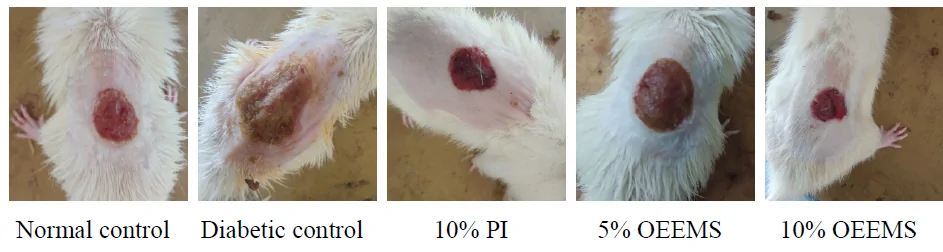
Representative photograph showing a wound on the 6^th^ day.

**Fig. (3):**
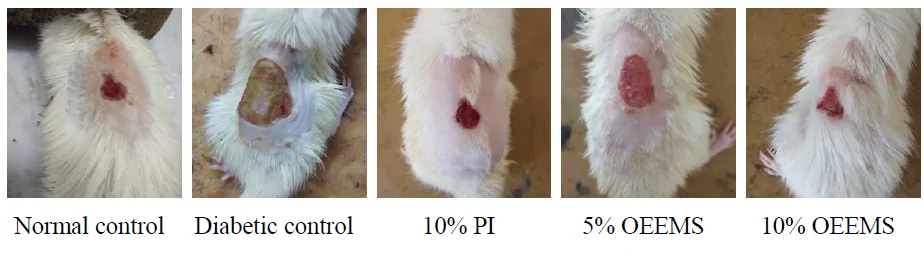
Representative photograph showing a wound on the 12^th^ day.

**Fig. (4):**
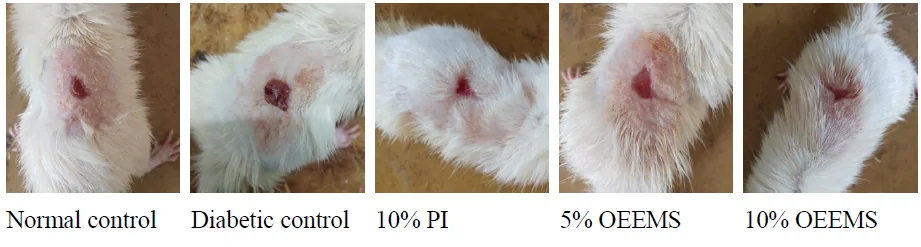
Representative photograph showing a wound on the 18^th^ day.

**Table 1:** Formula for simple ointment B.P.

**Ingredients**	**Composition**
Wool fat	5% w/w
Hard paraffin	5% w/w
White soft paraffin	85% w/w
Cetostearyl alcohol	5% w/w

**Table 2:** Results of preliminary phytochemical analysis of ethanol extract of *M. sativa* (EEMS).

**Compounds**	**EEMS**
Alkaloids	+
Carbohydrates	+
Flavonoids	+
Phenolic compounds	+
Phytosterols	+
Saponins	+
Tannins	-

**Note: ** (+) Present and (-) Absent.

**Table 3:** Effect of ethanol extract of *M. sativa* on blood glucose level (mmol/L) in excision wound model in diabetic rats.

**Group**	**^#^Blood Glucose Level (mmol/L)**		
	**Day 0**	**Day 10**	**Day 18**
Normal control	4.88 ± 0.16	5.1 ± 0.12	4.74 ± 0.08
Diabetic control	21.95 ± 0.36^a^	24.53 ± 0.33^a^	23.62 ± 0.42^a^
10% PI	19.62 ± 0.23	20.12 ± 0.13**	18.46 ± 0.19**
5% w/w OEEMS	18.81 ± 0.25	19.73 ± 0.21*	18.23 ± 0.21***
10% w/w OEEMS	19.92 ± 0.53	20.2 ± 0.44**	17.48 ± 0.34***

**Note:** ^#^Mean ± SEM (n = 6). Analysis of variance followed by Dunnett’s test with Diabetic control group; Significance represented as *p<0.05, **p<0.01, ***p < 0.001 and ^a^*p*<0.05 against Normal control. **Abbreviation:** OEEMS = Ointment of Ethanolic extract of *M. sativa*.

**Table 4:** Effect of *M. sativa* on body weight, food intake, and water intake in normal and alloxan-induced diabetic rats after 18 days of treatment.

**Groups**	**^#^Change in Body Weight (g)**		**Food Intake**	**Water Intake**
	**Initial**	**Final**	**(g/rat/day)**	**(ml/rat/day)**
Normal control	162.83 ± 5.97	186.16 ± 4.81	22.33 ± 2.36	29.66 ± 4.42
Diabetic control	171.33 ± 4.75	138.33 ± 3.91^a^	35.66 ± 3.46^a^	53.83 ± 5.62^a^
Povidone 10% w/w	170.5 ± 2.96	184.66 ± 3.63**	25.33 ± 4.56*	41.83 ± 6.28*
5% w/w OEEMS	165.5 ± 3.9	169.33 ± 2.88**	28.66 ± 4.13*	43.33 ± 5.35*
10% w/w OEEMS	175.66 ± 4.78	194.16 ± 3.85***	23.33 ± 3.21*	36.66 ± 3.81*

**Note:** ^#^Mean ± SEM (n = 6). Analysis of variance followed by Dunnett’s test with Diabetic control group; Significance represented as *p<0.05, **p<0.01, ***p < 0.001 and ^a^*p*<0.05 against Normal control. **Abbreviation:** OEEMS = Ointment of Ethanolic extract of *M. sativa*.

**Table 5:** Wound contraction area for excised wound in diabetic rats treated with ethanolic extract of *M. sativa.*

**Group**	**0 Day**	**2^nd^ Day**	**4^th^ Day**	**6^th^ Day**	**8^th^ Day**	**10^th^ Day**	**12^th^ Day**	**14^th^ Day**	**16^th^ Day**	**18^th^ Day**
**Normal Control**	546.66 ± 4.21	540.66 ± 3.16	518.66 ± 0.42	483.66 ± 0.61	454.66 ± 1.11	410.66 ± 0.42	357.66 ± 0.80	315 ± 1.12	277.33 ± 1.11	223.66 ± 1.30
		-1%	-5%	-11%	-16%	-24%	-34%	-42%	-49%	-59%
**Diabetic Control**	542.66 ± 1.42	539.66 ± 0.8	525.66 ± 0.61	502 ± 0.73	479 ± .34	456.33 ± 1.2	422.66 ± 0.84	388 ± 1.26	345.33 ± 0.98	292.33 ± 0.80
		-0.50%	-3%	-7%	-11%	-15%	-22%	-28%	-36%	-46%
**10% w/w PI**	541.33 ± 1.11	534.33 ± 0.61^c^	494 ± 1.26^c^	442 ± 0.73^c^	398.33 ± 0.61^c^	342.33 ± 1.2^c^	292 ± 1.15^c^	213.66 ± 0.95^c^	129.66 ± 0.80^c^	68.33 ± 1.29^c^
		-1.30%	-8%	-18%	-26%	-36%	-46%	-60%	-76%	-87%
**5% w/w OEEMS**	542.66 ± 0.92	536.66 ± 0.66^a^	521 ± 0.36^b^	486 ± 0.57^c^	447 ± 0.85^c^	421.33 ± 0.98^c^	376 ± 1.03^c^	346.66 ± 0.42^c^	299.33 ± 0.66^c^	248.33 ± 1.30^c^
		-1.10%	-4%	-10%	-17%	-22%	-30%	-36%	-44%	-54%
**10% w/w OEEMS**	543.66 ± 0.95	535.66 ± 0.61^b^	496.66 ± 0.66^c^	441.66 ± 0.61^c^	393.33 ± 0.98^c^	340.66 ± 0.98^c^	286 ± 1.40^c^	209.33 ± 0.84^c^	124 ± 0.73^c^	61 ± 1.91^c^
		-1.40%	-8%	-18%	-27%	-37%	-47%	-61%	-77%	-89%

Note: *Mean ± SEM (n = 6). Analysis of variance followed by Dunnett’s test with Diabetic control group; Significance represented as ^a^*p*<0.05, ^b^*p*<0.01, and ^c^*p* < 0.001. **Abbreviations: **OEEMS = ointment of ethanolic extract of *M. sativa*; SEM = standard error of the mean.

**Table 6:** Effect of OEEMS on skin epithelialization.

**Group**	**Time for Epithelialization (days)^#^**	**Decrease in Epithelialization (%)**
Normal control	22.66 ± 0.42	-
Diabetic control	26.83 ± 0.65^a^	-
Standard (PI 10% w/w)	19.83 ± 0.3***	26
5% w/w OEEMS	23.66 ± 0.49**	11.81
10% w/w OEEMS	19.16 ± 0.6***	28.58

**Note:** ^#^Mean ± SEM (n = 6). Analysis of variance followed by Dunnett’s test with Diabetic control group; Significance represented as ***p<0.001, and ^a^*p*<0.05 against Normal control. **Abbreviations: ** OEEMS = ointment of ethanolic extract of *M. sativa*; SEM = standard error of the mean.

**Table 7:** Fasting blood glucose level (mmol/L) of different treatment groups.

**Group**	**Blood Glucose (mmol/L)^#^**	
	**Day 0**	**Day 10**
Normal control	4.64 ± 0.19	5.07 ± 0.12
Diabetic control	18.19 ± 0.13	20.55 ± 0.26^a^
10% w/w PI	18.38 ± 0.19	19.21 ± 0.28*
5% w/w OEEMS	18.79 ± 0.17	19.13 ± 0.26*
10% w/w OEEMS	17.95 ± 0.12	18.64 ± 0.16**

**Note:** ^#^Mean ± SEM (n = 6). Analysis of variance followed by Dunnett’s test with Diabetic control group; Significance represented as *p<0.05, **p<0.01, and ^a^*p*<0.05 against Normal control. **Abbreviations: **OEEMS = ointment of ethanolic extract of *M. sativa*; SEM = standard error of the mean.

**Table 8:** Effect of OEEMS on wound breaking strength in an incision wound model.

**Group**	**Wound Breaking Strength (g)^#^**
Normal control	524.66 ± 6.5
Diabetic control	432.5 ± 4.47^a^
10% w/w PI	585.83 ± 3.33***
5% w/w OEEMS	498.83 ± 5.31***
10% w/w OEEMS	596.66 ± 2.29***

**Note:** ^#^Mean ± SEM (n = 6). Analysis of variance followed by Dunnett’s test with Diabetic control group; Significance represented as ***p<0.001, and ^a^*p*<0.05 against Normal control. **Abbreviations: ** OEEMS = Ointment of Ethanolic extract of *M. sativa*; SEM = standard error of the mean.

## Data Availability

The datasets generated during and/or analyzed during the current study are available from the corresponding author upon reasonable request.

## LIST OF ABBREVIATIONS

EEMS: Ethanolic Extract of *M. sativa*
PI: Povidone-iodine
